# All-optical modulation in Mid-Wavelength Infrared using porous Si membranes

**DOI:** 10.1038/srep30211

**Published:** 2016-07-21

**Authors:** Sung Jin Park, Ammar Zakar, Vera L. Zerova, Dimitri Chekulaev, Leigh T. Canham, Andre Kaplan

**Affiliations:** 1 Nanoscale Physics Research Laboratory, School of Physics and Astronomy, University of Birmingham, Edgbaston B15 2TT, United Kingdom; 2 pSiMedica Ltd. Malvern Hills Science Park, Geraldine Road, Malvern, WR14 3SZ, United Kingdom

## Abstract

We demonstrate for the first time the possibility of all-optical modulation of self-standing porous Silicon (pSi) membrane in the Mid-Wavelength Infrared (MWIR) range using femtosecond pump-probe techniques. To study optical modulation, we used pulses of an 800 nm, 60 femtosecond for pump and a MWIR tunable probe in the spectral range between 3.5 and 4.4 *μ*m. We show that pSi possesses a natural transparency window centred around 4 *μ*m. Yet, about 55% of modulation contrast can be achieved by means of optical excitation at the pump power of 60 mW (4.8 mJ/cm^2^). Our analysis shows that the main mechanism of the modulation is interaction of the MWIR signal with the free charge carrier excited by the pump. The time-resolved measurements showed a sub-picosecond rise time and a recovery time of about 66 ps, which suggests a modulation speed performance of ~15 GHz. This optical modulation of pSi membrane in MWIR can be applied to a variety of applications such as thermal imaging and free space communications.

Despite acute market demand there are currently no commercially available electro-optical modulators operating at Mid-Wavelength Infrared (MWIR) covering the wavelength range between 3.5 and 6 *μ*m. Yet, optical modulation is one of the key functionalities in optical telecommunications and thermal imaging devices for civilian and military applications. The present industrial applications rely entirely on mechanical modulators, which despite being mature and generally very reliable, have limitations of bulky dimensions, performance speed, susceptibility to g-forces and thirsty power consumption. The development of electro-optical modulators aims not only to improve these specifications but to introduce new capabilities, such as hyperspectral/multispectral and time-of-flight imaging, ultrafast non-uniform correction, rapidly activated antiflare protection, to otherwise well-developed MWIR technology.

A variety of optical modulation methods and their feasibility studies, including electro-optical^1^,^2^,^3^ and thermo-optical^4^, have previously been proposed. Among these, a technique based on the electron-hole plasma dispersion effect occupies a significant place because it promises greater spectral modulation depth and speed^1^. In this method optical properties change is induced by generation of the free charge carriers either by Franz-Keldysh effect based on application of electric field^5^ or by light absorption from an external source^6^. The latter method, so-called all-optical modulation, has attracted considerable research attention because it is deemed to provide a faster modulation speed and less noise, by avoiding conversion between electrical to optical signals. These advantages are being used to develop components for optical communications and network interconnects^7^,^8^, and also for dual-band filtering in IR multispectral imaging systems^9^,^10^,^11^, where a single camera detects the images of an object in multi-spectral bands. Numerous fundamental and technical achievements have been made so far, but their spectral capabilities cover mainly visible and near infrared (NIR) spectral bands^7^,^8^,^9^,^10^,^11^,^12^,^13^,^14^,^15^, while longer wavelengths remains out of technological reach.

Recently, research interest in all-optical modulation has expanded towards the longer wavelength of the MWIR range. The main motivation behind this is the potential benefits in free-space optical communications and multispectral IR imaging, with high performance in unfavourable atmospheric condition. Analogous with the previous semi-empirical approach^1^, theoretical calculations covering the range between 1 to 14 *μ*m^16^ demonstrated the feasibility of optical modulation for components made of crystalline Si, operating at MWIR and even longer wavelengths. The results showed that the change induced by the free carrier excitation to the refractive index is enhanced as the wavelength increases. Despite of an upsurge in the research interests in MWIR technology, only a few experimental studies have been done so far. All-optical modulation of quantum cascade lasers (QCL) as an optical source for the free-space optical communication has been studied at 4.72 *μ*m^17^, where the QCL is directly modulated by near IR pump beams (1.38 and 1.95 *μ*m). Optical modulation of crystalline silicon in the 2–2.5 *μ*m wavelength range has also been studied, which showed that the plasma dispersion effect is more prominent than for the traditional optical communication wavelengths (1.3 and 1.55 *μ*m)^18^, as expected from the theoretical prediction^16^. More recently, a modulation frequency of 23 kHz for 3.39 *μ*m was demonstrated on a novel silicon-on-lithium-niobate platform using Pockels effect^19^, while all-optical modulation of 50 MHz using a free-carrier absorption in germanium-on-silicon waveguides was demonstrated over the range of 2–3.8 *μ*m^20^. However, the majority of the experimental works covered only a few discrete wavelengths or a limited spectral band, while most of the MWIR spectrum still remains unexplored. In this work we demonstrate for the first time the possibility of all-optical modulation using pSi as an active material. The experiments were performed employing the femtosecond time-resolved pump-probe technique, with a probe wavelength tuned between 3.5 and 4.4 *μ*m, spanning almost the entire MWIR range. The time-resolved optical pump-probe technique is a suitable method because it allows investigation of all-optical modulation with a fast temporal resolution. It provides information of spectrally-resolved optical response and its temporal evolution, followed by pump excitation. The free-carriers generated by the pump beam, with energy just above the bandgap, induce a change to the optical properties, which are estimated by simultaneously measuring the intensity of the transmitted and reflected components of the probe beam. In the absence of heating, the time-resolved optical response measurements present the decay of the free-carriers, providing information about the modulation mechanism and speed. The main goal of this work is the first demonstration that self-standing pSi membranes can be used as active material in all-optical modulators. We show that pSi has unique natural properties of high transparency in MWIR while the transmittance at longer and shorter wavelengths is very low. That is, pSi can be considered as an MWIR passive band-pass filter. Moreover, we demonstrate that optical excitation at moderate fluence of about 4 mJ/cm^2^ modulates transmittance of the nearly transparent spectral window by about 55% accompanied by the corresponding increase of the absorbance and decrease of the reflectance. Our analysis suggests that the main mechanism of the modulation is attributed to the interaction of the MWIR radiation with the charge carriers excited by the pump. We suggest that the unique spectral properties of pSi, in combination with relatively low production costs and compatibility with well-established semiconductor devices manufacturing technologies, make it a good candidate for the development of electro-optical components for the IR technology. Moreover, pSi fabrication via anodisation has been demonstrated to be a scalable semiconductor cleanroom-compatible manufacturing process that is also amenable to on-chip integration of membranes^21^,^22^,^23^,^24^.

## Results and Discussion

**Figure 1:**
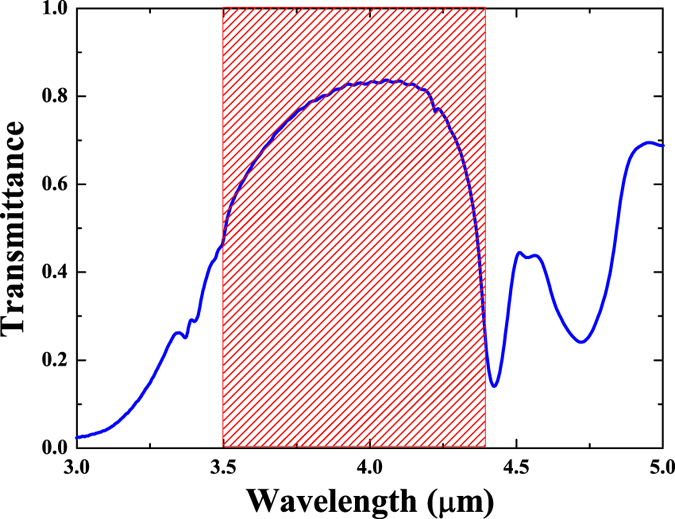
Transmittance, *T*_0_, of the 111 *μ*m-thick pSi membrane with 71% porosity measured by FTIR. The shaded area indicates the MWIR range (3.5–4.4 *μ*m).

Figure 1 shows the transmittance, *T*_0_, in the range between 3 and 5 *μ*m measured with Fourier Transform Infrared spectroscopy (FTIR) for 111 *μ*m-thick pSi membrane with 71% porosity. It is apparent that the transmittance strongly depends on the wavelength. In this work our main concern lies in the range within MWIR, especially 3.5–4.4 *μ*m, marked as a shaded area in Fig. 1, where there is a high transmission window of about ~84%, centred around 4.0 *μ*m. Both sides of this window are framed by peaks of strong absorption at 3.0, 4.4 and 4.7 *μ*m. This unique spectral feature of great optical contrast in transmittance creates an optical window transparent for MWIR, while nearby shorter and longer wavelength are blocked due to absorbance. We note that other samples of different thickness and porosity were also tested. However, the 111 *μ*m thick pSi membranes with 71% porosity showed nearly optimal spectral features–high contrast ratio between the transmission around 4.0 *μ*m and strong absorption due to surface adsorbates. It has been well-known that the transmittance decrease, on both sides of the transparent window, can be assigned to the absorption of impurities associated with the molecular vibration of Si-OH, Si-H, and O-Si-H stretching modes^25^,^26^,^27^,^28^ found on the surface. While in the bulk material these transitions are usually not observed because of their low optical density, in pSi they are very strong and stable because it has a very high surface-to-volume ratio. The absorption assignments of the FTIR results in Fig. 1 are detailed in Table 1. This arrangement of natural absorption peaks positions makes pSi a potential candidate for the design and development of passive band-pass MWIR filters. Moreover, in the following we show that the transparency of this window can be changed by an external optical excitation.

**Figure 2:**
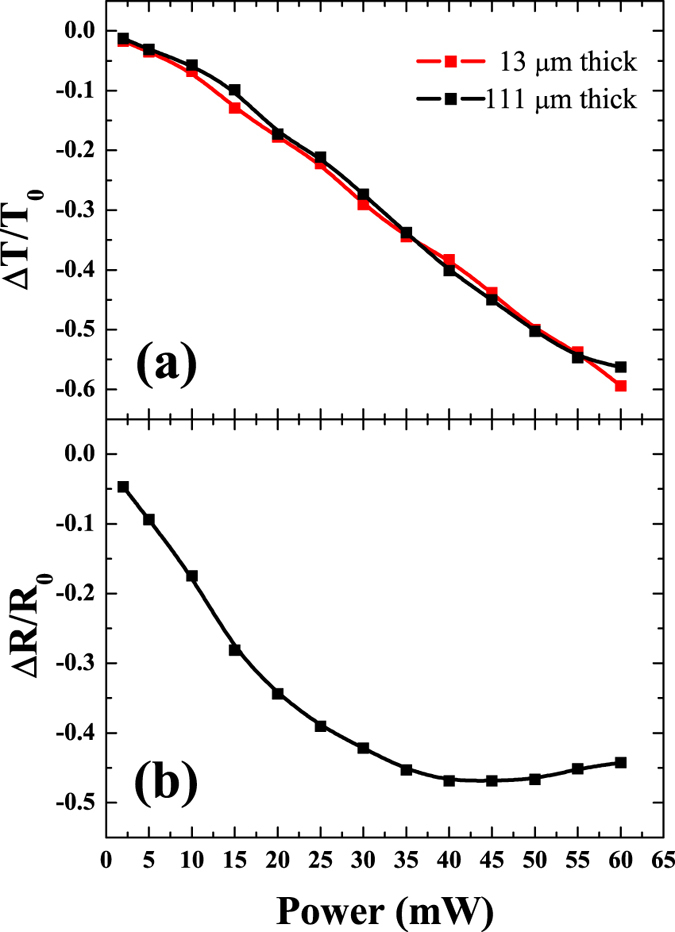
(**a**) Transmittance change, Δ*T*/*T*_0_, for the 4 *μ*m probe measured on the 13- and 111-*μ*m thick pSi membranes as a function of the pump beam power. (**b**) The reflectance change, Δ*R*/*R*_0_, as a function of the pump beam power at 4 *μ*m probe for the 111-*μ*m thick sample.

Figure 2(a) shows the fractional transmittance change (modulation contrast), Δ*T*/*T*_0_ (Δ*T*represents absolute change of the transmittance given by *T*_*p*_ − *T*_0_, where *T*_*p*_ is the transient transmittance after the pump excitation), for the 13 and 111 *μ*m-thick pSi membranes as a function of the pump power in the range between 2 and 60 mW, at 4 *μ*m probing wavelength, corresponding to the centre of the transparency window, and the 800 nm pump. These two thicknesses were chosen to compare the modulation contrast between relatively thin and thick samples. The measurements are made just after the zero delay between the pump and probe. The change in transmittance shows 1.3% at 2 mW and monotonically increases up to nearly 60% at 60 mW, corresponding to the range from 0.16 to 4.8 mJ/cm^2^. Although it was not experimentally tested, the observed trend suggests that even higher modulation could be achieved at higher pumping powers. It is noteworthy that the transmittance change is independent of sample thickness. This observation implies that the active region responsible for the transmittance change induced by the optical excitation occurs at near the surface region. Indeed, it is known from the previous studies of pSi optical properties that the 800 nm pump beam intensity decays by a factor of 1/*e* within the distance of ~4.6 *μ*m from the surface, which is thinner than both samples^29^. On the other hand, Fig. 2(b) shows the fractional change of the reflectance, Δ*R*/*R*_0_, recorded simultaneously with Δ*T*/*T*_0_ and measured for the thicker membrane. Here we observed an initial strong decrease followed by saturation. This combination of negative changes for both reflectance and transmittance implies that the optically excited membrane became strongly absorptive. The most likely mechanism of 4 *μ*m probe signal absorption is interaction with the charge carriers excited by the pump. To examine this suggestion, we derived the experimental optical density, *α*_*probe*_*d*, (where *α*_*probe*_ is the absorption coefficient of the 4 *μ*m probe and *d* is the sample thickness), using data shown in Fig. 2(a,b) and separately measured values of the transmittance, *T*_0_, and the reflectance, *R*_0_, (see Methods).

**Figure 3:**
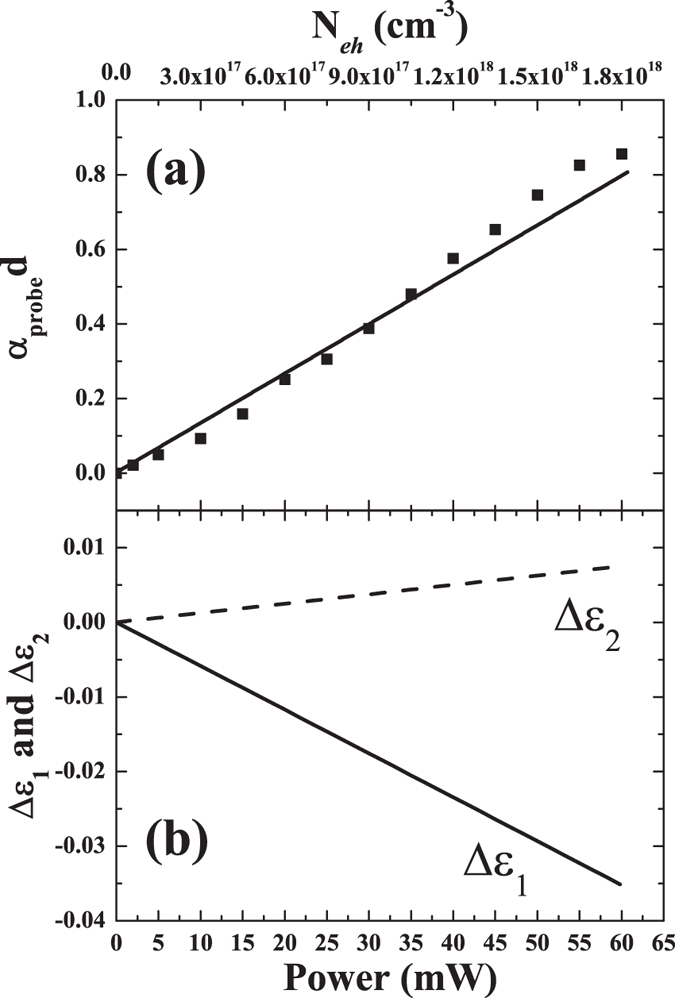
(**a**) The experimentally determined (black squares) and calculated (solid line) optical density, *α*_*probe*_*d*, of the 4 *μ*m probe as a function of the pump power. (**b**) The real and imaginary parts, Δ*ε*_1_ and Δ*ε*_2_, respectively, of the dielectric function changes as a function of the pump power. The corresponding excited carrier concentration, *N*_*eh*_, is also displayed on the upper *x*-axis on the top panel.

The experimentally estimated optical density (dotted data) is shown in Fig. 3(a). To get more insight into the physics underlying the observed modulation and to retrieve quantitative information about the excited charge carriers, we modelled the optical response of the membrane, namely its real and imaginary parts of the dielectric function, using the Maxwell-Garnett model, modified to include optical response of the excited charge carrier described by the Drude theory (see Methods). The estimated changes in the dielectric function are presented in Fig. 3(b) as a function of the pump power. It has been shown previously^29^ that the Drude theory provides a good approximation and can be used to estimate the excited charge carrier concentration, *N*_*eh*_, and the damping rate, Γ. We note that the estimate of phenomenological Γ does not directly reveal the nature of the charge carrier scattering process and we have left this out of the arguments in this work. Our purpose here is to provide an acceptable estimate of the carrier concentration, *N*_*eh*_, and the damping rate, Γ. Under assumption that the number of the excited free carriers linearly depend on the pumping power, the excited carrier concentration, was calculated using the fluence, *F*, corresponding to the each pump power at 4 *μ*m probe beam wavelength (see Methods), and is also displayed on the upper x-axis on the top panel. Figure 3(a) shows that calculations assuming the linear dependence of on pumping power, and keeping the damping rate, Γ, constant at the value of 3 × 10^14 ^s^−1^, provide a reasonable agreement with the experimentally determined optical density.

The calculated free-carrier density on the samples surface spans between 6 × 10^16^ and 1.8 × 10^18^ cm^−3^ for the average pulse power range covering values from 2 to 60 mW. This procedure confirms that, immediately after the excitation, the change of optical response in band around 4 *μ*m can be explained by the contribution from the excited free charge carriers.

**Figure 4:**
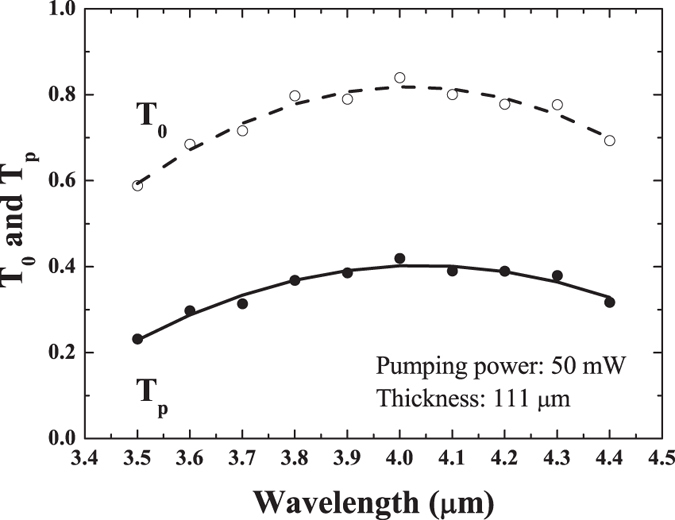
*T*_*p*_ is the transmittance of the 111-*μ*m thick sample under excitation by the 50 mW pump as a function of the wavelength, measured by the tunable probe beam. The dashed and solid lines are a guide to the eye. *T*_0_ is the transmittance of the probe beam without the pump.

Another important matter in question is the change to the transmittance spectrum induced by the pump, as it determines the efficiency of modulation as a function of the wavelength. Figure 4 shows the spectral dependence of the transmittance at a pump power of 50 mW, covering the wavelength range from 3.5 to 4.4 *μ*m, where the transmission fractional change of 50% was observed. Surprisingly, the observed nearly flat response of *T*_*p*_ as a function of the wavelength, resembles the free carrier absorption spectrum in this wavelength range in doped bulk Si. It has been known for a long time that a sharp increase of the absorbance, with nearly quadratic dependence, occurs at the wavelengths longer than 5 *μ*m which is preceded by a part of the spectrum which is nearly independent of wavelength^30^. (We note that *T*_0_ on Fig. 4 slightly differs on the edges from the FTIR spectrum shown on Fig. 1. The discrepancy arises because *T*_0_ on Fig. 4 shows a convolution of a rather broad MWIR femtosecond laser pulse spectrum with that shown in Fig. 1.)

**Figure 5:**
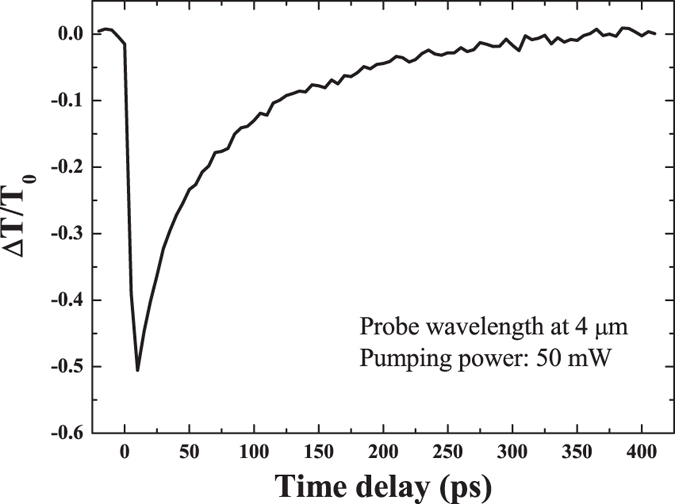


To develop the optical modulation device, the rise and recovery (decay) times are important parameters. Figure 5 shows the fractional transmittance change at 4.0 *μ*m and the 50 mW pump power as a function of the delay time. It can be seen that the rise time is as fast as the sub-picosecond resolution of the measurements. Such a fast response provides additional support to our suggestion that the optical response is governed by the free carriers excitation. On the other hand, the decay time at which the intensity of the transmission change drops by a factor of 1/e was 66 ps. Thus, pSi has the potential to operate at 15 GHz modulation speed in spectrum filtering devices. Although the exact mechanism of the carrier concentration decay remains debatable, we compare our result to those ultrafast measurements published previously. Our findings are rather similar to the works which estimated a fast component of 100 ps attributed to the bimolecular recombination^31^,^32^. Yet, an additional slow microsecond long component observed there and attributed to the radiative recombination involving surface states is absent in our work, because its excitation requires a pump with the wavelength shorter than 800 nm. On the other hand, the fast decay of 1 ps shown elsewhere^33^,^34^ was not present in any of our measurement. In cited works the fast component was attributed to the carrier trapping by dangling bond states. In our work the dangling bonds are not likely to have such important impact as the samples are passivated by oxides and hydroxides molecules.

We have demonstrated that a self-standing porous Si membrane could be used as an optical intensity modulator in MWIR in the range between 3.5 and 4.4 *μ*m. FTIR measurements showed that a porous Si membrane has a high transmittance spectral window centred around 4 *μ*m and surrounded from both sides of the spectrum by strongly absorptive vibrational modes of molecular impurities concentrated on the surface. We have shown that the transparency of this window can be modulated by external optical excitation achieving around 55% contrast at the pump power of 60 mW (4.8 mJ/cm^2^). Our analysis demonstrates that the main mechanism affecting the transparency of the window can be attributed to and modelled by the response of the excited charge carriers. We also provide estimates of the carrier concentration and the damping time. We have measured the transient properties of the transmittance response and shown that it has a subpicosecond rise time and a decay of 66 picoseconds. Thus, pSi can be used in modulators operating with a speed of above ten GHz. Overall, we conclude that pSi provides advantages not found in other materials, such as a natural bandpass window in MWIR, fast response and a higher absorption coefficient for the pump than in bulk silicon. We suggest further investigation to improve modulation contrast and achieve nearly absolute change of the transmittance from the transparent to the fully opaque state. As possible directions for development one might consider affecting the band structure by, for example, artificially introduced stress, which might enhance the absorption. Alternatively, growth of pSi as stratified multilayer structures might enhance both filtering and modulation characteristics.

## Methods

### Preparation of porous silicon membranes

The porous silicon membrane samples were prepared by electrochemical anodisation of a (100) silicon wafer (*B*-doped, 5–15 mΩcm). The electrolyte was a mixture of methanol and 40% HF in a ratio 1:1 and a current density was 30 mA/cm^2^. The porous layer was detached from the underlying substrate by applying a 120 mA/cm^2^ pulse (10 s) before it was removed from the electrolyte. With precisely controlled anodisation time, different thickness of porous Si layer can be produced. The free-standing membranes were then rinsed in methanol and dried in air. The pores naturally passivated in ambient environment creating a protective cladding consisting of a mix of oxide and hydroxide molecules. More details on the fabrication can be found in ref. 35. A sponge-like porous structure was observed in scanning electron micrographs. The porosity of 71% was estimated using gravimetric analysis. The representative Scanning Electron micrographs are shown in the Supplementary Information.

### Optical measurements

To measure time-resolved pump-probe reflectivity and transmission change, Δ*R*/*R*_0_ and Δ*T*/*T*_0_, measurements were performed using a Coherent laser system delivering 60 fs pulses at a repetition rate of 1 kHz, 800 nm central wavelength and about 3W average pulse power. The main beam was split into two unequal parts, with a weaker beam of 120 mW used as the pump. The remnant beam was directed to feed an optical parametric amplifier (OPA) to generate the 150 fs probe beam in the MWIR range covering the wavelength between 3.5 to 5 *μ*m. The incidence angles of the probe and pump were 24° and 60°, respectively. The choice of the angles was made to separate the beams paths spatially, so no stray light of the pump will reach the detectors, and to set the reflectance detector as close to the normal as the physical dimension of the detector will allow. The pump power was tuned by means of an attenuator consisting of a linear polariser and a half-wave polarisation rotator. To measure the transmittance and reflectance change simultaneously, the same type of InSb photoconductive detectors (Hamamatsu P6606-320) were used. The time delay between the pump and probe beams was scanned using a computer-controlled delay stage consisting of a retroreflector mounted on a precision translation stage. The schematic diagram of the experimental set-up is displayed in the Supplementary Information. For the FTIR measurements of the transmittance at the ground state, *T*_0_, a Bruker FTIR Hyperion microscope was employed. The FTIR measurements were performed with an unpolarised beam in air at room temperature.

### Optical response calculation

The dielectric function given below by Eq. 1 is based on the Maxwell-Garnett effective medium approximation modified to include the Drude optical response of the pump excited charge carriers^36^.


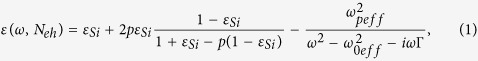


where


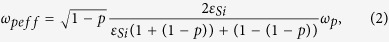



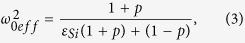


and


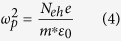


where *ε*_0_ is the dielectric function of the bulk Si at the ground state, *p* is the membrane porosity, Γ is the carrier damping rate, *ω* is the frequency of the probe radiation, *ω*_0*eff*_ and *ω*_0*peff*_ are the effective resonant and plasma frequencies, respectively, *ω*_*p*_ is the plasma frequency of the free space, *N*_*eh*_ is the concentration of the free-carriers on the surface, *ε*_0_ is vacuum permittivity, *e* is electron charge, and m* is the optical effective mass of 0.17. *N*_*eh*_ is assumed to decay along the membrane depth coordinate, *z*, as exp(−*α*_*pump*_*z*), where *α*_*pump*_ is absorption coefficient of the 800 nm pump given in the literature^29^.

The dielectric function in Eq. 1 and the frequencies in Eqs 2 and 3 were obtained by following the derivation of equations given by Sihvola^37^ which describes the case of isolated metallic particles in a non-conducting medium. Here we investigate an inverse case of non-conductive cavities embedded in a conductive medium. The validity of the obtained expressions was checked at the limits of *p* = 0 and *p* = 1 corresponding to the cases for bulk silicon and air, respectively. The Drude term of in Eq. 1 includes the damping rate Γ = 3 × 10^14 ^s^−1^ which is assumed to be constant as a function of the carrier concentration and the coordinate z. Using a linear approximation, the concentration on the surface, at *z* = 0, was calculated according to 
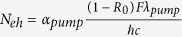
, where *F* and *λ* are the pump fluence and wavelength, respectively. The second order process in pSi has a two-photon absorption coefficient of less than 1 cm/GW. Thus, it can be neglected because over the range of investigated intensities the pump generates through this process almost an order of magnitude lower carrier concentration than in the linear one^38^. However for the higher pump intensities it needs to be included. With the set of the aforementioned parameters we used Eq. 1 to calculate the dielectric function and to work out the imaginary part of the refractive index, *k*, and the absorption coefficient, *α*_*probe*_, given below by Eqs 5, 6, respectively,


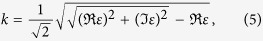


and


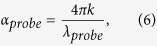


The obtained results were iteratively fitted to the experimental data of the optical density gained from the measurements of the reflectance, *R*, and transmittance, *T*, using the following relation:


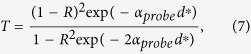


where d* = *d*/cos*θ*_*i*_, with *θ*_*i*_ representing the angle of incidence of the probe beam. *T* and *R* were determined from the measurements of *T*_0_ = 0.80 and *R*_0_ = 0.13, and with Δ*R*/*R*_0_ and Δ*T*/*T*_0_ obtained in the pump probe measurements.

## Additional Information

**How to cite this article**: Park, S. J. *et al*. All-optical modulation in Mid-Wavelength Infrared using porous Si membranes. *Sci. Rep.* **6**, 30211; doi: 10.1038/srep30211 (2016).

## Supplementary Material

Supplementary Information

## Figures and Tables

**Table 1:** Absorption peak assignments in the mid-wavelength infrared range (3–5 *μ*m) from refs 25,26,27,28.

**Vibrational mode**	**Absorption peak position in** ***μ*****m (cm**^**−1**^)
O_1_SiH_3_	4.63 *μ*m (2160 cm^−1^)
O_2_SiH_2_	4.52 *μ*m (2210 cm^−1^)
O_3_SiH_1_	4.41 *μ*m (2270 cm^−1^)
SiH	4.79 *μ*m (2087 cm^−1^)
SiH_2_	4.74 *μ*m (2108 cm^−1^)
SiH_3_	4.67 *μ*m (2142 cm^−1^)
Si-OH	3 *μ*m (3330 cm^−1^)
