# Supplementary material for: All-optical modulation in Mid-Wavelength Infrared using porous Si membranes
Source: Sci Rep. 2016 Jul 21;6:30211. doi: 10.1038/srep30211 (PMC4954954; doi:10.1038/srep30211)
Supplement: Supplementary Information [file srep30211-s1.pdf]

Supplementary Information

All-optical modulation in Mid-Wavelength Infrared using porous Si membranes

Sung Jin Park[1], Ammar Zakar[1], Vera L. Zerova[1] , Dimitri Chekulaev[1*],
Leigh T. Canham[1,2] & Andrey Kaplan[1]

[1]Nanoscale Physics Research Laboratory, School of Physics and Astronomy,
University of Birmingham, Edgbaston B15 2TT, United Kingdom
[2]pSiMedica Ltd. Malvern Hills Science Park, Geraldine Road, Malvern, WR14 3SZ,
United Kingdom

1. Scanning Electron Microscope (SEM) images for pSi membranes.

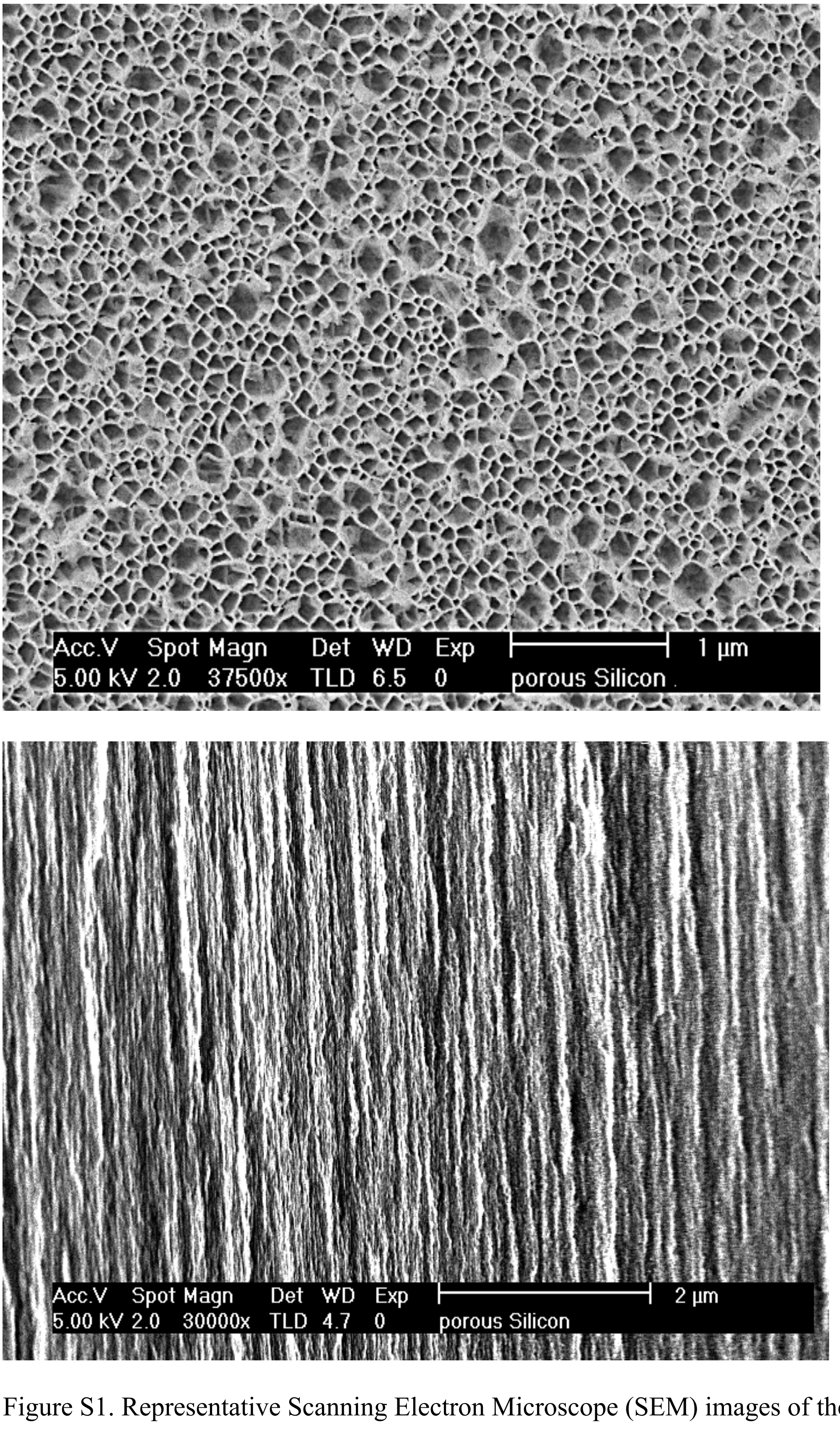

Figure S1. Representative Scanning Electron Microscope (SEM) images of the 13 um-thick pSi. The upper image: a top view; the bottom image: a side view.

2. Schematic diagram of femtosecond pulsed laser pump probe system

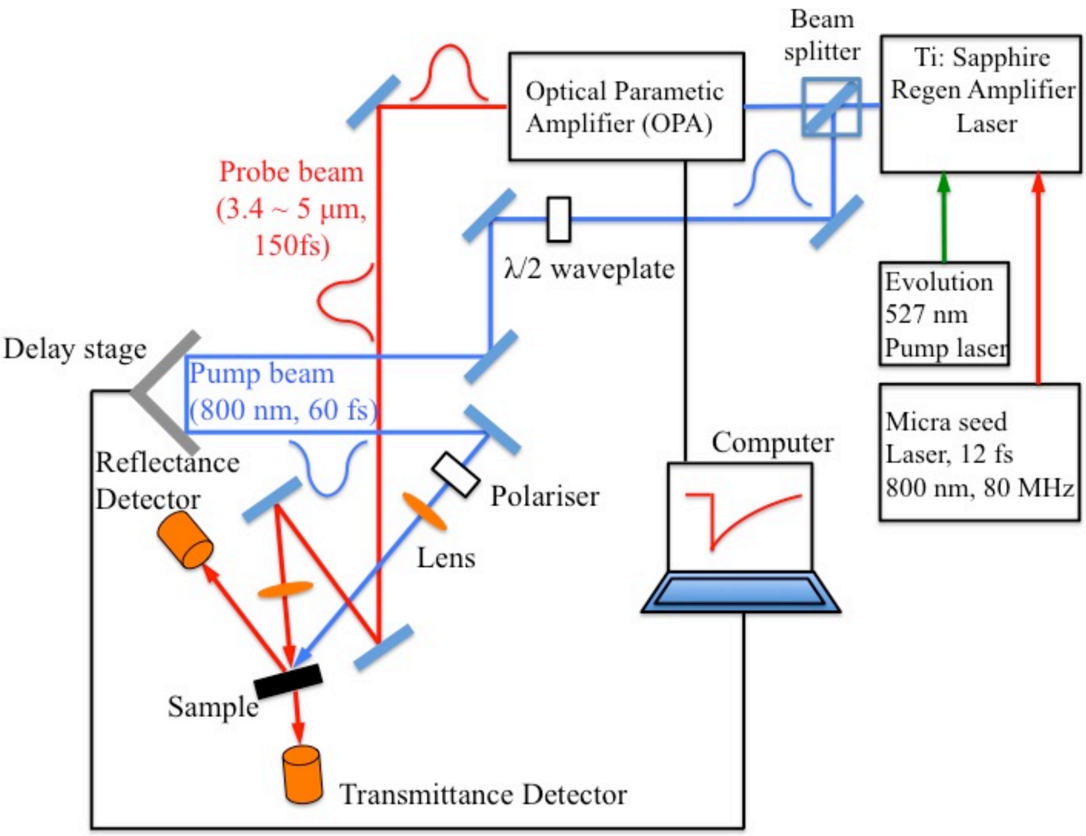

Figure S2. Schematic diagram of the pump-probe experimental set-up. The delay stage controls the arrival/delay time of the probe pulse (red line) with respect to the pump pulse (blue line). The time resolution of the system is defined by the probe beam pulse duration of 150 fs. The wavelength of the probe beam can be set within 3.4 – 5 micrometre range. The reflected and transmitted components of the probe beam are detected by Hamamatsu P6606-320 detectors connected to lock-in amplifiers.
